# TaWRKY115 enhances cold tolerance by weakening the expression of TaMYB4 on CBFs in common wheat

**DOI:** 10.1093/nsr/nwag087

**Published:** 2026-02-09

**Authors:** Jinyuan Liu, Yaoyao Jiang, Lei Zhao, Qianhui Xi, Anxin He, Fangyu Xiang, Jiaqi Li, Lingran Zhang, Congwei Sun, Ning Zhang, Jian Yang, Feng Chen

**Affiliations:** State Key Laboratory of High-Efficiency Production of Wheat-Maize Double Cropping, College of Agronomy, Henan Agricultural University, Zhengzhou 450046, China; State Key Laboratory for Quality and Safety of Agro-products, Key Laboratory of Biotechnology in Plant Protection of MARA, Key Laboratory of Green Plant Protection of Zhejiang Province, Institute of Plant Virology, Ningbo University, Ningbo 315211, China; State Key Laboratory of High-Efficiency Production of Wheat-Maize Double Cropping, College of Agronomy, Henan Agricultural University, Zhengzhou 450046, China; State Key Laboratory of High-Efficiency Production of Wheat-Maize Double Cropping, College of Agronomy, Henan Agricultural University, Zhengzhou 450046, China; State Key Laboratory of High-Efficiency Production of Wheat-Maize Double Cropping, College of Agronomy, Henan Agricultural University, Zhengzhou 450046, China; State Key Laboratory of High-Efficiency Production of Wheat-Maize Double Cropping, College of Agronomy, Henan Agricultural University, Zhengzhou 450046, China; State Key Laboratory of High-Efficiency Production of Wheat-Maize Double Cropping, College of Agronomy, Henan Agricultural University, Zhengzhou 450046, China; State Key Laboratory of High-Efficiency Production of Wheat-Maize Double Cropping, College of Agronomy, Henan Agricultural University, Zhengzhou 450046, China; State Key Laboratory of High-Efficiency Production of Wheat-Maize Double Cropping, College of Agronomy, Henan Agricultural University, Zhengzhou 450046, China; State Key Laboratory of High-Efficiency Production of Wheat-Maize Double Cropping, College of Agronomy, Henan Agricultural University, Zhengzhou 450046, China; State Key Laboratory for Quality and Safety of Agro-products, Key Laboratory of Biotechnology in Plant Protection of MARA, Key Laboratory of Green Plant Protection of Zhejiang Province, Institute of Plant Virology, Ningbo University, Ningbo 315211, China; State Key Laboratory of High-Efficiency Production of Wheat-Maize Double Cropping, College of Agronomy, Henan Agricultural University, Zhengzhou 450046, China

**Keywords:** common wheat, cold tolerance, *TaWRKY115*, *TaMYB4*, *TaCBF*, *TaSP1*

## Abstract

Low temperature as one of the most important abiotic stresses has caused serious wheat production loss worldwide. We identified *TaWRKY115* affecting cold tolerance in wheat by integration of genome-wide association study and RNA sequencing. Overexpression and CRISPR/Cas9-mediated gene-editing revealed *TaWRKY115* positively modulating cold tolerance in wheat. Transcription factor *TaSP1* negatively regulates the cold tolerance of wheat by binding to the promoter of *TaWRKY115* in cold-sensitive haplotypes. TaWRKY115 inhibited TaMYB4 at both protein and transcription levels. BSMV-mediated silenced wheat plants and EMS mutants of *TaMYB4* possessed increased wheat cold tolerance. We further revealed that *TaMYB4* repressed expressions of the *TaCBF* family through binding to their promoters. BSMV-mediated silenced wheat plants and EMS mutants of *TaCBF12d* exhibited decreased cold tolerance in wheat. This study brought forth a new model TaSP1-TaWRKY115-TaMYB4, regulating wheat cold tolerance through the *TaCBF* pathway and provided valuable cold tolerance genes for wheat breeding programs.

## INTRODUCTION

Low temperature is one of the most important abiotic stresses to seriously threaten plant growth. When plants are exposed to low temperature stress, the fluidity of cell membranes and the conformation of proteins are altered [1]. Therefore, plants evolved various strategies to cope with extremely low temperature, such as changes in membrane structure, accumulation of antifreeze substances, and activation of the ROS scavenging system [2]. Dissection of the molecular mechanisms of plant cold stress will be valuable in order to improve crop cold tolerance via molecular breeding strategies.

Wheat is one of the three staple food crops widely grown in temperate regions and provides the majority of energy source for humans and livestock [3]. Low temperature inhibits the growth and development processes of wheat plants and thus causes serious yield losses [4]. To date, some genetic loci modulating cold tolerance have been detected, but only *Fr-1* and *Fr-A2* were identified to show a stable effect in wheat [5]. *Fr-1* was assumed to be a pleiotropic effect of *Vrn-1* controlling wheat vernalization, and *Fr-A2* is possibly a copy number variation of a CBF (C-repeat binding factor) gene on 5A [6,7]. In addition, two *ICE1*-like genes (*TaICE41* and *TaICE87*) have been isolated from a cDNA library prepared from cold-treated wheat aerial tissues and showed an intimate association with cold tolerance, possibly through activating CBF gene expression in wheat [8]. Recently, we revealed that *TaPGK* [9] and *TaSnRK1α* [10] played key roles in regulating wheat cold tolerance through the pyruvate acid and jasmonic acid pathways, respectively. Therefore, identification of important cold response genes and dissection of their molecular mechanisms are of great significance for the improvement of wheat cold tolerance.

As immobile organisms, plants have developed a series of defense mechanisms to adapt to cold stress in their long evolutionary process. Many studies have reported that the CBF family played important roles in the process of cold acclimation in multiple plants [11,12]. As transcription factors (TFs), CBF specifically binds to the promoter containing CRT/DRE *cis*-acting element of target genes, and activates the expression of COR (cold-responsive) genes after cold stress [12–14]. Up to now, various transcription factors have been reported to regulate CBF expression at low temperature, including ICE [15], MYB TFs [16], CAMTA TFs [17,18] and PIFs [19,20].

As a large TF family in plants, MYB family genes have been reported to profoundly regulate cold tolerance through the CBF pathway [21]. In apple, MdMYB88 and MdMYB124 improved cold tolerance by activating *MdCBF3* via regulation of *MdCCA1* expression [22]. In *Rosa multiflora, RmMYB108* improved plant cold tolerance by upregulating the CBF cascade, and overexpression of *RmMYB108* significantly enhanced cold tolerance in *Arabidopsis* [23]. In rice, OsMYB3R-2 regulated *OsCPT1* expression in the DREB/CBF pathway and promoted proline synthesis under cold stress, improving cold tolerance [24]. In *Arabidopsis*, MYB15 acted as a negative regulator of cold stress by controlling *CBF3* expression [16]. In wheat, *TaMYB56-B* possibly mediated some genes involved in DREB1/CBF signal transduction, and *TaMYB56-*overexpressed plants displayed increased cold tolerance [25].

With the development of high-throughput sequencing technology in recent years, genome-wide association study (GWAS) became an effective strategy to identify the important genes regulating complex traits in wheat [26,27]. For example, we cloned a *TaHST1L* for wheat tiller angle [27] and a *TaMTB* for wheat yellow mosaic virus (WYMV) resistance [28] through multiple strategies containing GWAS. In this study, we identified an important wheat cold tolerance gene, *TaWRKY115*, by the integration of GWAS and transcriptome sequencing, and demonstrated its function by genetic transformation. We further found that the expression of *TaWRKY115* was regulated by the TF TaSP1, and TaWRKY115 strongly interacted with TaMYB4 that is a transcriptional repressor of the *TaCBF* family. Our results discovered valuable genes and proposed a new insight into cold tolerance signaling pathway in wheat.

## RESULTS

### TaWRKY115 was significantly associated with wheat cold stress

**Figure 1.**
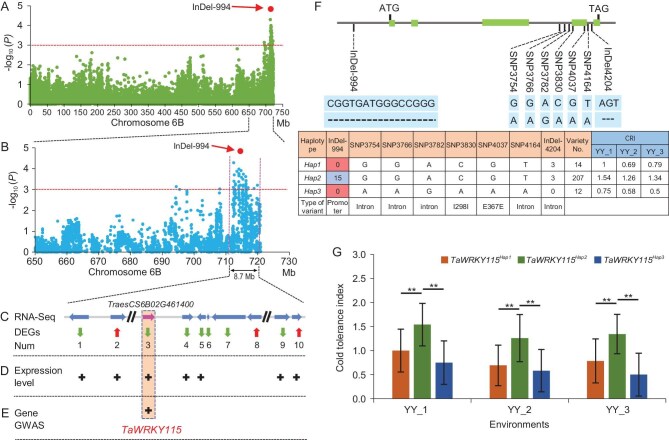
Mapping and cloning of wheat cold tolerance gene *TaWRKY115*. (A) Manhattan plot for wheat seedling cold tolerance identified by GWAS on Chromosome 6B. (B) Local Manhattan plot surrounding the peak on 6B. In (A) and (B), the red line represents the significance threshold, and the arrows indicate the most significant SNP, which was located in *TaWRKY115* promoter. (C) Differentially expressed genes within the candidate interval revealed by RNA-Seq. The red arrow represents up-regulated expression and the green represents down-regulated expression of genes induced by cold stress. (D) Analysis of expression patterns of candidate genes. The genes highly expressed in roots, stems and leaves are shown as ‘**+**’. (E) The results of Gene-Based GWAS by using the markers developed based on the sequence variation sites within candidate genes. (F) The gene structure, polymorphism loci and haplotypes of *TaWRKY115*. The green blocks represent exons. YY_1: 2021_Yuanyang; YY_2: 2022_Yuanyang Repeat 1; YY_3: 2022_Yuanyang Repeat 2. (G) Comparison of cold response index (CRI) among different haplotypes in the association panel.

To identify important genetic loci modulating wheat cold tolerance, we performed a genome-wide association study (GWAS) analysis for cold response index (CRI) over 2 years in 233 wheat accessions previously genotyped using the Wheat 660 K SNP array (Fig. S1A) [9,10]. GWAS results revealed an important genetic locus on 6B for CRI in 2 years (Figs 1A and S1B; Table S1). Physical position analysis showed that most of the significant SNPs on 6B clustered into an 8.7-Mb interval (712.2–720.9 Mb) in the genome of Chinese Spring (IWGSC RefSeq v2.0) (Fig. 1B; Table S1). Regional distribution of significant SNPs on 6B revealed that the 8.7 Mb could be further divided into three sub-intervals (712.25–712.99 Mb, 714.07–717.96 Mb and 720.11–720.95 Mb) covering 110 genes (Fig. S1C; Table S2). RNA sequencing results of wheat plants before and after cold stress indicated that 10 of these 110 genes were significantly induced by cold stress (Figs 1C and S2A; Table S3), and 7 of them were highly expressed in roots, stems, or leaves of seedlings (Figs 1D and S2B). Next, we sequenced these 7 genes in 233 wheat accessions by Wheat Pan800K Gene Chip and obtained a total of 102 variants (Table S4). The gene-based GWAS after adding 102 variants into the Wheat 660 K SNP array showed that only variants associated with *TraesCS6B02G461400* (annotated as WRKY transcription factor-like protein) were significantly detected (Fig. 1A, 1B and 1E; Tables S1 and S4). Haplotype analysis of these 7 genes showed that only *TraesCS6B02G461400* was significantly associated with cold tolerance (Figs 1F, 1G and S2C). Thus, *TraesCS6B02G461400* named as *TaWRKY115* was selected for further analysis (Fig. S3).

### *TaWRKY115* positively regulated wheat cold tolerance

**Figure 2.**
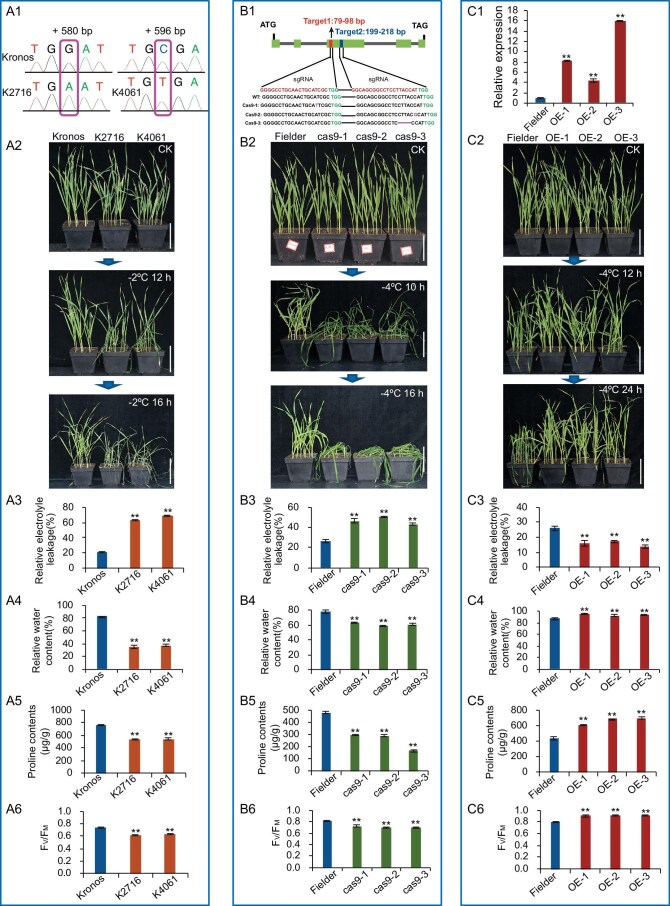
Functional verification of *TaWRKY115*. (A1) Mutation sites identified by sequencing of two Kronos mutant lines of *TaWRKY115*. The mutation sites are marked with purple boxes. (A2) Phenotype comparison between the wild type Kronos and mutant plants (*n* = 10 plants per replicate) before and after cold stress treatment. Scale bars, 10 cm. (A3-A6) The relative electrolyte leakage (A3), relative water content (A4), proline content (A5) and F_V_/F_M_ ratios (A6) between mutants and the wild-type Kronos plants after cold stress treatment. (B1) The mutation types of three *TaWRKY115-*edited lines created by Crispr-cas9 gene editing. The green letters indicate PAM sequences and red letters mean editing sites. (B2) Phenotype comparison between Fielder and *TaWRKY115-*edited lines (*n* = 10) before and after cold stress treatment. Scale bars, 10 cm. (B3–B6), The relative electrolyte leakage (B3) and relative water content (B4), proline content (B5) and F_V_/F_M_ ratios (B6) between Fielder and *TaWRKY115*-silenced lines after cold stress treatment. (C1) The relative expression between Fielder and *TaWRKY115* overexpression lines. (C2) Phenotype comparison between Fielder and *TaWRKY115* overexpression lines (*n* = 10) before and after cold stress treatment. Scale bars, 10 cm. (C3–C6) The relative electrolyte leakage (C3) and relative water content (C4), proline content (C5) and F_V_/F_M_ ratios (C6) between Fielder and *TaWRKY115* overexpression lines. * and ** indicate significant differences determined by Student’s *t*-test at *P* < 0.05 and *p* < 0.01, respectively. The experiments were conducted with three biological replicates.

To verify the function of *TaWRKY115*, we screened two mutants (K2716 and K4061 with a premature stop codon of *TaWRKY115*) from the EMS-mutagenized tetraploid wheat Kronos library (Figs 2A1 and S4A), and then backcrossed them into BC_2_ lines with wild-type (WT). After cold stress, both K2716 and K4061 BC_2_ lines were more sensitive to low temperature and exhibited more serious drooping and wilting than WT (Figs 2A2 and S4B). In addition, both mutants showed significantly increased relative electrolyte leakage (Fig. 2A3) and decreased relative water content (Fig. 2A4), decreased proline content (Fig. 2A5) and decreased F_V_/F_M_ ratios (Fig. 2A6) compared with WT. It suggested that mutation of *TaWRKY115* sharply reduced cold tolerance in tetraploid wheat.

Subsequently, we performed a CRISPR/Cas9-mediated gene editing system to create *TaWRKY115*-edited mutants in Fielder (Fig. 2B1). The wheat mutation sites were confirmed by sequencing using Hi-TOM (Table S5). Then three independent M_0_ plants were self-crossed twice into M_2_ generation, and mutation sites were further confirmed by Sanger sequencing (Fig. S4C). After cold stress, all of the three *TaWRKY115*-edited lines exhibited more serious drooping and wilting (Fig. 2B2) and possessed significantly increased relative electrolyte leakage (Fig. 2B3), decreased relative water content (Fig. 2B4), decreased proline content (Fig. 2B5) and decreased F_V_/F_M_ ratios (Fig. 2B6) compared with WT.

We next constructed a wheat LGY-OE vector containing the full-length cDNA of *TaWRKY115* and transformed it into the cold-sensitive wheat cultivar Fielder for overexpression (OE). Three positive T_0_ lines were self-crossed into T_2_ generation and showed high expression of *TaWRKY115* as detected by qRT-PCR (Fig. 2C1). After cold stress, three *TaWRKY115*-OE transgenic lines exhibited less drooping and wilting (Figs 2C2 and S4D) and possessed significantly decreased relative electrolyte leakage (Fig. 2C3), increased relative water content (Fig. 2C4), increased proline content (Fig. 2C5) and increased F_V_/F_M_ ratios (Fig. 2C6) compared with WT. It suggested that overexpression of *TaWRKY115* sharply enhanced cold tolerance in wheat plants. In summary, *TaWRKY115* positively regulated cold tolerance in wheat.

### The expression of *TaWRKY115^Hap2^* was downregulated by *TaSP1*

Sequencing analysis showed that *TaWRKY115* possessed eight allelic variants resulting in three haplotypes (Fig. 1F). Wheat accessions harboring *TaWRKY115^Hap2^* exhibited the highest CRI among these three haplotypes (*P* < 0.01) (Fig. 1G), while 88.8% of all test accessions harbored *TaWRKY115^Hap2^*, suggesting that the cold-sensitive allele *TaWRKY115^Hap2^* is predominant in the Yellow and Huai Valleys of China.

**Figure 3.**
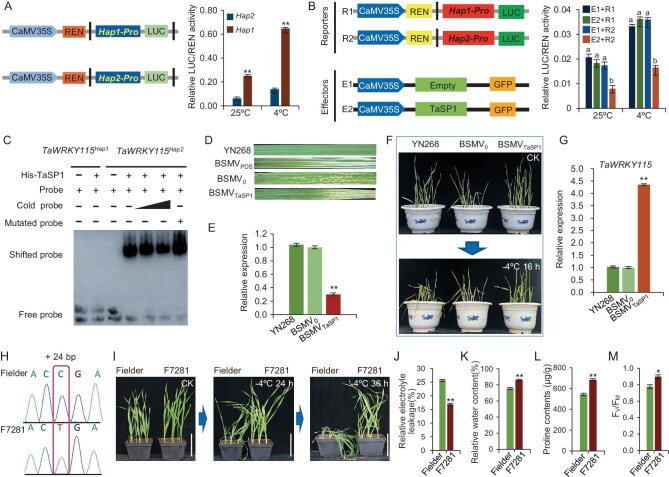
TaSP1 suppressed the expression of *TaWRKY115^Hap2^*. (A) Determination of promoter activity in different *TaWRKY115* haplotypes under different temperatures. (B) TaSP1 suppressed the promoter activity of *TaWRKY115^Hap2^* under normal and cold conditions. *TaSP1* were co-infiltrated with either *TaWRKY115^Hap1^* or *TaWRKY115^Hap2^* promoter in *Nicotiana benthamiana* leaves. The Luc/Ren ratio indicated the transcriptional activity of different promoters by effectors. Significant differences were calculated among different combinations using Tukey’s multiple comparison tests. (C) EMSA assay revealed TaSP1 bound to the GC-box in the *TaWRKY115^Hap2^* promoter. Bound probe and shift probe were detected with anti-streptavidin-horseradish peroxidase (HRP), respectively. (D) Leaf phenotypes of VIGS experiment. (E) Relative expression level of *TaSP1* in YN268, BSMV_0_ and BSMV_TaSP1_ plants. (F) Phenotype comparison of YN268, BSMV_0_ and BSMV_TaSP1_ plants (*n* = 6) before and after cold treatment. (G) The expression level of *TaWRKY115* in BSMV_TaSP1_ plants. (H) Mutation site identified by sequencing of *TaSP1* mutants (F7281). The mutation site is marked with a purple box. (I) Phenotype comparison between Fielder and F7281 plants (*n* = 10 plants per replicate) before and after cold stress. Scale bars, 10 cm. (J–M) The relative electrolyte leakage (J), relative water content (K), proline content (L) and F_V_/F_M_ ratios (M) between mutants and the wild-type plants after cold treatment. * and ** indicate significant difference determined by Student’s *t*-test at *p* < 0.05 and *p* < 0.01, respectively. The experiments were conducted with three biological replicates.

Sequence variants showed that *TaWRKY115^Hap2^* notably had a 15-bp insertion (InDel-994) in its promotor region compared with the other two haplotypes (Fig. 1F; Table S6) while other variants are either synonymous mutation or in introns. Therefore, we speculated that the 15-bp insertion possibly led to differential cold tolerance between *TaWRKY115^Hap2^* and others. A transient expression assay by dual-luciferase showed that the transcription activity of *TaWRKY115^Hap2^* promoter was significantly lower than that of *TaWRKY115^Hap1^* under both normal and cold conditions (Fig. 3A). Furthermore, the expression level of *TaWRKY115^Hap^^1^* was significantly higher than that with *TaWRKY115^Hap2^* under both normal and cold conditions (Fig. S5A). Sequence analysis revealed that the 15-bp insertion covered a GC-box (GGGCGG) (Fig. S5B) that was previously reported to be bound by a transcription factor (TF) *SP1* (zinc finger family protein) [29,30]. qRT-PCR results showed that the expression level of *TaSP1* (*TraesCS6A02G199800*) was significantly upregulated in Yunong 268 (YN268) harboring *TaWRKY115^Hap2^*under cold stress (Fig. S5C). Dual-LUC assay showed that *TaSP1* significantly suppressed the expression of *TaWRKY115^Hap2^* under both normal and cold conditions (Fig. 3B). Moreover, DNA electrophoretic mobility shift assay (EMSA) showed that *TaSP1* could specially bind to the GC-box in the promoter of *TaWRKY115^Hap2^* (Fig. 3C). Subsequently, we silenced *TaSP1* by virus-induced gene silencing (VIGS) in YN268 (Fig. 3D and E). Results showed that BSMV_TaSP1_-silenced plants showed significantly stronger cold tolerance compared with controls after cold stress (Fig. 3F). qRT-PCR results showed that the expression level of *TaWRKY115* was significantly increased in BSMV_TaSP1_-silenced plants compared with WT (Fig. 3G). We next screened a *TaSP1* mutant (F7281) with premature stop codon from the EMS-mutagenized hexaploid wheat Fielder library (Fig. 3H). After cold stress, the F7281 plants showed less drooping and wilting (Fig. 3I), and possessed significantly decreased relative electrolyte leakage (J), increased relative water content (K), increased proline content (L) and increased F_V_/F_M_ ratios (M) compared with WT. These results suggested that *TaSP1* inhibited *TaWRKY115^Hap2^* expression to negatively regulate wheat cold tolerance.

### TaWRKY115 as a transcription factor inhibited the expression of TaMYB4

**Figure 4.**
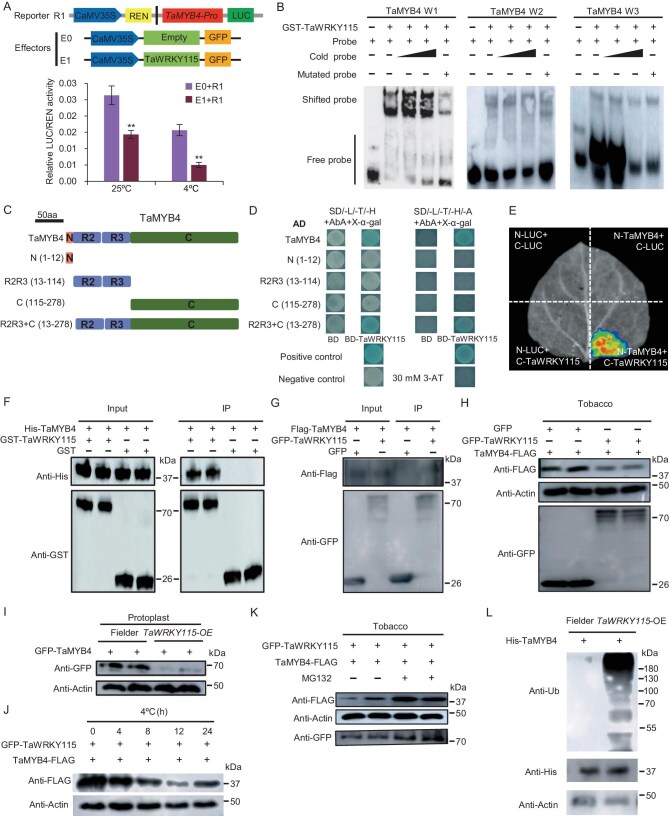
TaWRKY115 interacted with TaMYB4 at both DNA-protein and protein–protein levels. (A) Dual-luciferase assay in *N. benthamiana* leaves indicated that TaWRKY115 suppressed the promoter activity of *TaMYB4* under both normal and cold stress treatment. * and ** indicate significant differences determined by Student’s *t*-test at *p* < 0.05 and *p* < 0.01, respectively. The experiments were conducted with three biological replicates. (B) EMSA assay revealed TaWRKY115 bound to the W-boxes in the *TaMYB4* promoter. Bound probe and shift probe were detected with anti-streptavidin-horseradish peroxidase (HRP), respectively. (C) Schematic representation about different domains of TaMYB4. (D) Interaction of TaWRKY115 and TaMYB4 in Y2-H assay. The negative control represents vectors pGBKT7-Lam and pGADT7-T, the positive control represents vectors pGBKT7-53 and pGADT7-T as per Clontech’s user manual. SD/–T–L–H, selective medium lacking Trp, Leu, His; SD/–T–L–H–A, selective medium lacking Trp, Leu, His and Ade. Chromogenic substrate X-α-Gal was added to the medium; 3-amino-1,2,4-triazole (3-AT) was used as the inhibitor. AbA: Aureobasidin A. (E) Firefly luciferase complementation imaging (LCI) assay confirmation of TaWRKY115 and TaMYB4 interaction in *N. benthamiana* leaves. N-LUC or N-TaMYB4 were co-transformed with C-LUC or C-TaWRKY115, respectively. Empty vectors were used as controls. (F) Verification of the physical interaction between TaWRKY115 and TaMYB4 by pull-down *in vitro*. For Input and IP, samples from adjacent channels (left two or right two) were from the same tube. (G) Verification of the physical interaction between TaWRKY115 and TaMYB4 by Co-IP in wheat protoplast. (H) TaWRKY115 significantly inhibited the protein abundance of TaMYB4 with (+) or without (−) TaWRKY115 in tobacco leaves. An empty vector (GFP) was used as a negative control and anti-actin was used as a loading control. (I) Transient expression of pJIT163-Ubi-hGFP: TaMYB4 in WT Fielder and *TaWRKY115*-OE wheat leaf protoplasts. Actin was used as an internal reference. (J) Cold stress enhanced TaWRKY115 inhibiting TaMYB4 at the protein level. The TaMYB4 protein levels were detected using an anti-FLAG antibody. (K) TaWRKY115 inhibited the accumulation of TaMYB4 through the 26S proteasome system. (L) The ubiquitination level of TaMYB4 was detected in *TaWRKY15-OE* plants but not in Fielder. Anti-actin and anti-His antibodies were used as controls.

Phylogenetic analysis indicated that *TaWRKY115* belongs to the WRKY TF family (Fig. S3) that was widely reported to bind to the W-box (T)TGAC(C/T) sequence [31]. Subcellular localization in wheat protoplasts showed that the TaWRKY115 was mainly localized in the nucleus, plasma membrane and cytoplasm (Fig. S6A). TaWRKY115 exhibited self-transactivation activity in yeast cells (Fig. S6B). To identify target genes regulated by *TaWRKY115*, we sequenced the transcriptome of *TaWRKY115*-OE and WT plants. Results showed that 3669 differentially expressed genes (DEGs) were detected between WT and *TaWRKY115*-OE plants (Table S7). Furthermore, we employed DNA affinity purification sequencing (DAP-seq) to determine genome-wide binding sites, and identified 5754 targets of TaWRKY115 (Fig. S7A and Table S8). Combined analysis of RNA-seq and DAP-seq revealed an overlap of 201 target genes (Fig. S7B and Table S9), and 5 of them were speculated to respond to cold based on GO (Gene Ontology) term analysis (Table S10). Then *TaMYB4* (*TraesCS2B02G399200*) was selected since the MYB family as transcription factors play important roles in regulating cold stress in plants [21,^32^]. qRT-PCR showed that the expression level of *TaMYB4* was significantly reduced in the *TaWRKY115*-OE plants, especially after cold stress (Fig. S8A and S8B). Notably, three TTGACC (W-box) motifs were identified in the *TaMYB4* promoter region (Fig. S8C). Dual-luciferase assay in tobacco leaves revealed TaWRKY115 greatly repressed the expression of the luciferase reporter gene driven by the *TaMYB4* promoter under both normal and cold conditions (Fig. 4A). We next performed an EMSA, and results showed that TaWRKY115 bound to all three motifs (Fig. 4B). These results indicated that TaWRKY115 as a transcription factor inhibited the expression of *TaMYB4* at the transcription level.

### TaWRKY115 interacted with TaMYB4 *in vivo* and *in vitro*

To further explore its potential mechanism as a protein, we used TaWRKY115 as a bait to screen interaction proteins by yeast two-hybrid (Y2H) in a wheat cDNA library. Coincidentally, TaMYB4 was present in the screened results. The interaction of TaWRKY115 and TaMYB4 was further verified by Y2H (Fig. 4C and D). In addition, both R2R3 and C-terminal regions of TaMYB4 are necessary to interact with TaWRKY115 (Fig. 4C and D). The interaction was also confirmed in tobacco leaves by firefly luciferase complementation imaging (LCI) assay (Fig. 4E). Microscale thermophoresis (MST) assay results showed that the recombinant protein TaMYB4-His specifically interacted with TaWRKY115-GST but did not interact with the purified protein OsWRKY76-GST (Fig. S9A and S9B). *In vitro* pull-down results further revealed TaWRKY115 interacting with TaMYB4 (Fig. 4F). Co-immunoprecipitation (Co-IP) assay in wheat protoplasts confirmed that TaMYB4 could be co-immunoprecipitated with TaWRKY115 (Fig. 4G). These results showed that TaWRKY115 interacted with TaMYB4 both *in vitro* and *in vivo*. Immunoblot results in tobacco showed that the protein level of TaMYB4 gradually decreased with time under cold treatment (Fig. S10A).

### TaWRKY115 inhibited TaMYB4 at both protein and transcriptional levels

To analyze the influence of TaWRKY115 on TaMYB4 at the protein level, we co-transformed 35S: TaMYB4-FLAG with 35S: GFP or 35S: TaWRKY115-GFP into tobacco cells for instantaneous expression, respectively. Western blot results showed that TaWRKY115 reduced the protein abundance of TaMYB4 (Fig. 4H). Similarly, western blot in wheat protoplast indicated that the protein level of TaMYB4 was significantly decreased in the *TaWRKY115*-OE plants using an anti-GFP antibody compared with WT (Fig. 4I). Furthermore, we found that low temperature (4°C) promoted the inhibition of TaMYB4 protein level by TaWRKY115 (Fig. 4J). Co-expression of 35S: TaMYB4-FLAG with 35S: TaWRKY115-GFP and MG132 showed that TaWRKY115 reduced the protein level of TaMYB4 through the 26S proteasome system (Fig. 4K). We next detected the ubiquitination level of TaMYB4 in *TaWRKY15*-OE plants but not in WT (Fig. 4L). Subcellular localization in wheat protoplasts showed that TaMYB4 was localized in the nucleus (Fig. S10B). Integration of transcription and protein results indicated that TaWRKY115 inhibited TaMYB4 as a dual-suppression through protein–protein interaction and transcription regulation.

### *TaMYB4* negatively modulated wheat cold tolerance

**Figure 5.**
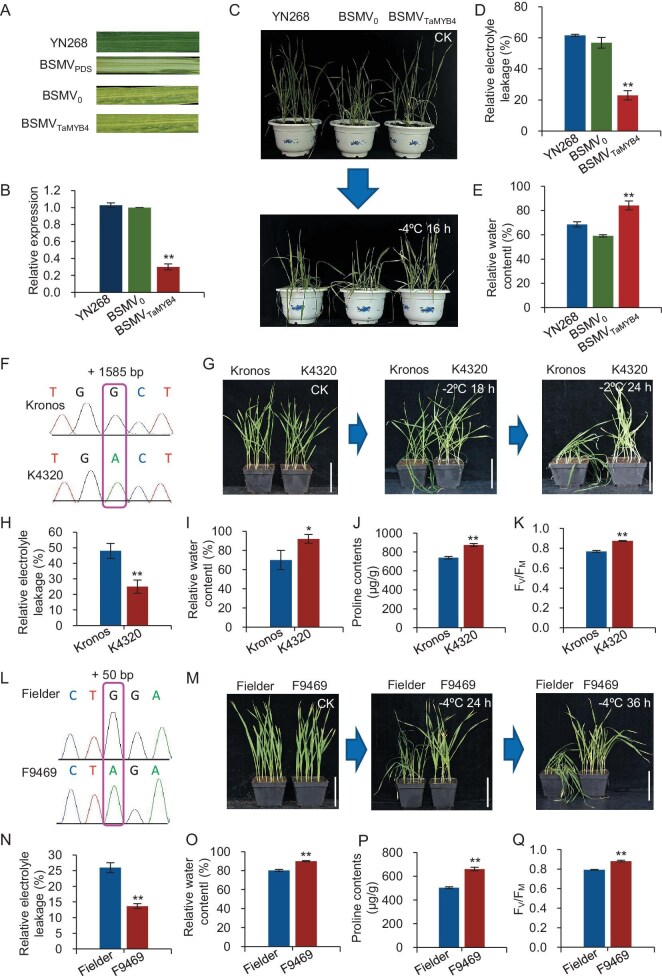
TaMYB4 negatively regulated wheat cold tolerance. (A) Leaf phenotypes in VIGS experiment. YN268 is non-silenced plant used as the negative control. BSMV_PDS_ was used as a positive control to monitor the time course of VIGS. BSMV_0_ acted as the viral control. BSMV_TaMYB4_: *TaMYB4*-silenced plant. (B) Relative expression level of TaMYB4 in WT, BSMV_0_, BSMV_TaMYB4_. (C) Phenotypic comparison between YN268, BSMV_0_ and BSMV_TaMYB4_ plants (*n* = 6) before and after cold stress. (D and E) Determination of relative electrolyte leakage (D) and relative water content (E) between YN268, BSMV_0_ and BSMV_TaMYB4_ plants after cold stress. (F) Mutation site marked with purple box was identified by sequencing of *TaMYB4* tetraploid wheat mutant (K4320). (G) Phenotype comparison between Kronos and K4320 lines (*n* = 10) before and after cold stress. Scale bars, 10 cm. (H–K) Determination of relative electrolyte leakage (H) and relative water content (I), proline content (J) and F_V_/F_M_ ratios (K) between Kronos and K4320 plants after cold stress. (L) Mutation site marked with purple box was identified by sequencing of *TaMYB4* hexaploid wheat mutant (F9469). (M) Phenotype comparison between Fielder and F9469 plants (*n* = 10 plants per replicate) before and after cold stress. Scale bars, 10 cm. (N–R) The relative electrolyte leakage (N), relative water content (O), proline content (P) and F_V_/F_M_ ratios (Q) between mutants and the wild-type plants after cold stress. * and ** indicate significant differences determined by Student’s *t*-test at *P* < 0.05 and *p* < 0.01, respectively. The experiments were conducted with at least three biological replicates.

To verify the function of *TaMYB4* modulating wheat cold stress, we silenced *TaMYB4* in YN268 by VIGS (Fig. 5A). qRT-PCR showed that the transcription level of *TaMYB4* was significantly reduced in *TaMYB4-*silenced plants (Fig. 5B). After cold stress, the *TaMYB4*-silenced plants showed stronger cold tolerance (Fig. 5C), significantly decreased relative electrolyte leakage (Fig. 5D) and significantly increased relative water content (Fig. 5E) compared with controls.

We next screened a mutant K4320 with a premature stop codon of *TaMYB4* from the EMS-mutagenized tetraploid wheat Kronos library (Figs 5F and S11), and backcrossed K4320 into BC_2_ lines with WT. After cold stress, the K4320 BC_2_ lines had less drooping and wilting (Fig. 5G), significantly decreased relative electrolyte leakage (Fig. 5H), increased relative water content (Fig. 5I), increased proline content (Fig. 5J) and increased F_V_/F_M_ ratios (Fig. 5K) compared with WT. We also screened a mutant F9469 with a premature stop codon of *TaMYB4* from the EMS-mutagenized hexaploid wheat Fielder library (Fig. 5L). After cold stress, the F9469 plants showed less drooping and wilting (Fig. 5M), significantly decreased relative electrolyte leakage (Fig. 5N), increased relative water content (Fig. 5O), increased proline content (Fig. 5P) and increased F_V_/F_M_ ratios (Fig. 5Q) compared with WT. These results strongly suggested that *TaMYB4* negatively regulated wheat cold tolerance.

### TaMYB4 directly bound to the *TaCBF12d* promoter to inhibit its expression

**Figure 6.**
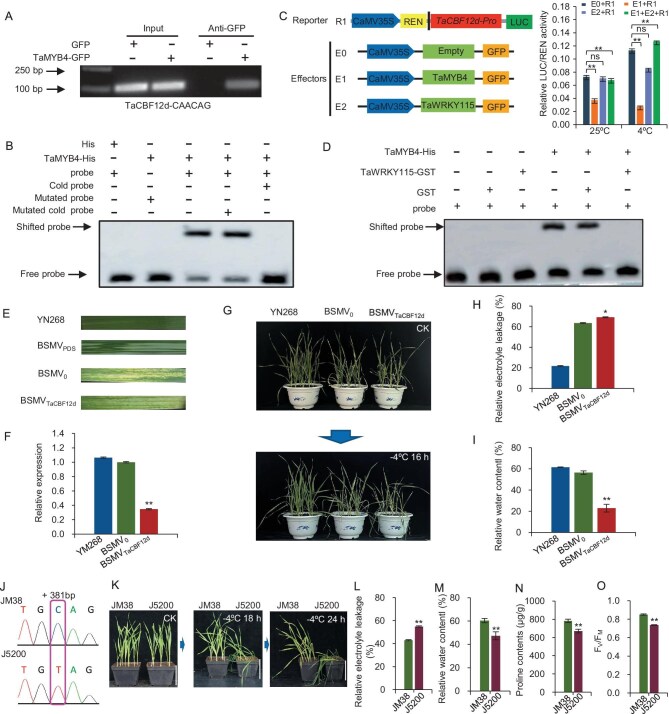
TaMYB4 directly binds to the promotor of *TaCBF12d* to downregulate its expression. (A) ChIP-PCR result showed that TaMYB4 directly targeted the *TaCBF12d* promoter. The promoter fragment containing CAACAG was immunoprecipitated with antibody against GFP. (B) TaMYB4 interacted with *TaCBF12d* promoter by EMSA. (C) The regulatory relationships of *TaCBF12d* by TaWRKY115 and TaMYB4 were detected by dual-luciferase assay in *N. benthamiana* leaves at 4 and 25°C, respectively. (D) EMSA assay revealed the effect of TaWRKY115 on TaMYB4 binding to the -CAACAG- binding site in the *TaCBF12d* promoter. Bound probe and shift probe were detected with anti-streptavidin-horseradish peroxidase (HRP), respectively. (E) Leaf phenotypes of VIGS experiment. (F) Relative expression level of *TaCBF12d* in YN268, BSMV_0_ and BSMV_TaCBF12d_ plants. (G) Phenotype comparison of YN268, BSMV_0_ and BSMV_TaCBF12d_ plants (*n* = 6) before and after cold stress. (H and I) Determination of relative electrolyte leakage (H) and relative water content (I) in YN268, BSMV_0_ and BSMV_TaCBF12d_ plants before and after cold stress. (J) Mutation site marked with purple box was identified by sequencing of *TaCBF12d* mutant line (J5200). JM38, hexaploid wheat cultivar Jimai38. (K) Phenotype comparison between the wild-type JM38 and J5200 plants (*n* = 10 plants per replicate) before and after cold stress. Scale bars, 10 cm. (L–O) The relative electrolyte leakage (L), relative water content (M), proline content (N) and F_V_/F_M_ ratios (O) between wild-type JM38 and J5200 plants after cold stress. * and ** indicate significant differences determined by Student’s *t*-test at *p* < 0.05 and *p* < 0.01, respectively. The experiments were conducted with three biological replicates.

To dissect the mechanism of *TaMYB4* participating in cold tolerance, we performed RNA-seq analysis in wheat plants before and after cold stress, and identified 37 differentially expressed CBF genes 14 of which were significantly induced by low temperature (Table S11). Further analysis showed that 7 (*TaCBF6a, TaCBF6b, TaCBF6d, TaCBF9d, TaCBF10a, TaCBF12d* and *TaCBF14d*) of these 14 genes were highly expressed in roots, stems or leaves of seedlings (Fig. S12A). Analysis of promoter sequences showed that all of these 7 CBF genes contained MYB-binding motifs (-CAACAG- or -TAACAG-) (Fig. S12B). qRT-PCR results indicated that expression levels of all these 7 genes were significantly increased in *TaMYB4*-silenced plants compared with controls (Fig. S12C), suggesting that the CBF family genes were possibly downregulated by TaMYB4. Due to the relatively higher expression level of *TaCBF12d* among 7 genes (Fig. S12C), we performed ChIP-PCR for *TaCBF12d* and results revealed that an expected DNA fragment containing the predicted MYB binding site was amplified using a *TaCBF12d*-specific primer (Figs 6A and S13A). MST assay further illustrated that TaMYB4 specially bound to the *TaCBF12d* promoter containing a CAACAG motif *in vitro* (Fig. S13B). Moreover, we performed an EMSA and found that the purified recombinant His-TaMYB4 fusion protein specifically bound to the CAACAG motif in the *TaCBF12d* promoter (Fig. 6B). Taken together, these results suggested that *TaMYB4* inhibited the expression of *TaCBF12d* by directly binding to its promoter.

### The TaWRKY115-TaMYB4 model was involved in TaCBF pathway regulating wheat cold tolerance

To illustrate the three-level regulatory relationship of *TaWRKY115, TaMYB4* and *TaCBF12d*, we employed a dual-luciferase assay system in tobacco. Consequently, *TaMYB4* but not *TaWRKY115* significantly suppressed the expression level of *TaCBF12d* (Fig. 6C). However, *TaMYB4* with the addition of *TaWRKY115* alleviated the inhibition of *TaCBF12d* (Fig. 6C). Additionally, cold stress enhanced the expression of *TaCBF12d* through the addition of *TaMYB4* and *TaWRKY115* (Figs 6C and S14A), suggesting that the expression of *TaCBF12d* was released possibly due to the interaction of TaWRKY115 and TaMYB4. EMSA results showed that TaWRKY115 significantly inhibited TaMYB4 binding to the *TaCBF12d* promoter (Fig. 6D). qRT-PCR showed that the expression of *TaCBF12d* was significantly upregulated in *TaWRKY115*-OE plants but was significantly downregulated in *TaWRKY115* mutant plants (Fig. S14B and S14C). Taken together, these results indicated that *TaMYB4* inhibited the expression of *TaCBF12d* by directly binding to its promoter, while TaWRKY115 as a dual-suppressor of TaMYB4 could weaken *TaCBF12d* repression from TaMYB4.

To identify the role of *TaCBF12d* in modulating wheat cold stress, we silenced *TaCBF12d* in YN268 by VIGS (Fig. 6E and F). After cold stress, the *TaCBF12d*-silenced plants exhibited serious drooping and wilting (Fig. 6G), significantly increased relative electrolyte leakage (Fig. 6H) and decreased relative water content (Fig. 6I). We subsequently screened a mutant J5200 with a premature stop codon of *TaMYB4* from the EMS-mutagenized hexaploid wheat Jimai38 (JM38) library (Fig. 6J). After cold stress, the J5200 plants exhibited serious drooping and wilting (Fig. 6K), and possessed significantly increased relative electrolyte leakage (Fig. 6L), decreased relative water content (Fig. 6M), decreased proline content (Fig. 6N) and decreased F_V_/F_M_ ratios (Fig. 6O) compared with WT. These results suggested that *TaCBF12d* positively regulated wheat cold tolerance. Hence, *TaMYB4* possibly inhibits expressions of the *TaCBF* family by binding to their promoters to indirectly regulate wheat cold tolerance.

In summary, we proposed a *TaSP1*-*TaWRKY115-TaMYB4-TaCBF* cascade to modulate wheat cold tolerance. Cold stress increasingly induced the expression of *TaSP1* in wheat plants. Cold-tolerant haplotype *TaWRKY115^Hap1^* that could not be downregulated by *TaSP1* under cold stress inhibited TaMYB4 as a dual-suppressor, thereby causing the normal expression of the TaCBF family and ultimately exhibiting cold tolerance (Fig. S15). However, cold-sensitive haplotype *TaWRKY115^Hap2^* that was downregulated by *TaSP1* under cold stress did not efficiently inhibit TaMYB4, and thus enhanced the suppression of the TaCBF family by TaMYB4, finally resulting in cold sensitivity (Fig. S15).

## DISCUSSION

Cold stress is a major factor that limits the geographical distribution of crops around the world and has resulted in a huge reduction in crop yield [33]. In wheat, cold damage frequently occurs in major wheat production regions worldwide, and annually causes serious yield loss in many countries [34]. Therefore, identification of important cold stress genes is of great significance in order to improve cold tolerance in wheat by pyramid breeding. However, due to the large size and high duplication of the hexaploid wheat genome, it is difficult to directly clone key wheat genes only through a single traditional mapping technique. In this study, we identified an important cold tolerance gene, *TaWRKY115*, by the integration of GWAS, transcriptome sequencing, tissue-specific gene expression, and sequencing. Through screening downstream target genes and interaction proteins of TaWRKY115, we uncovered a transcription factor TaMYB4, which is a homologous gene of *OsMYB4* in rice. We proved that TaMYB4 can interact with TaWRKY115 at both DNA–protein and protein–protein levels, and further verified its negative role in regulating cold tolerance. Additionally, we found that *TaCBF12d* positively modulated wheat cold tolerance. Therefore, the discovery of these three wheat genes will be beneficial for improving wheat cold tolerance via gene pyramiding breeding.

The *TaWRKY115* gene was annotated as a WRKY transcription factor-like protein in WheatOmics (http://wheatomics.sdau.edu.cn/). Previous studies showed that WRKY TFs were involved in the CBF pathway to regulate plant responses to low temperature. Mutation of *AtWRKY34* may enhance the expression of CBFs in *Arabidopsis*, thus improving cold tolerance [35]. In Bermudagrass, CdWRKY2 positively regulated cold stress by targeting the *CdCBF1* promoter and activating *CdCBF1* expression [36]. In wheat, *TaWRKY19* bound to *DREB2A* promoter and activated the expression of *DREB2A*, which enhanced cold tolerance [37]. However, the current understanding of the regulatory mechanism of WRKY TFs in wheat cold tolerance is still very limited. In this study, the overexpression of *TaWRKY115* significantly improved the cold tolerance of wheat plants, while EMS mutants and CRISPR/Cas9-mediated knockout plants of *TaWRKY115* showed more sensitive phenotypes to cold stress, providing new evidence for WRKY TFs to participate in wheat cold stress. In addition, our data showed that a 15-bp InDel in the promoter of *TaWRKY115* gene affected its expression, thereby changing the cold tolerance of wheat plants, which was consistent with previous reports [38]. Cultivars without the 15-bp insertion (*TaWRKY115^Hap^^1^*and *TaWRKY115^Hap^^3^*) showed significantly higher *TaWRKY115* expression and stronger cold tolerance than those with the 15-bp insertion (*TaWRKY115^Ha^^p2^*). Furthermore, the expression of *TaWRKY115^Hap2^* was efficiently suppressed by *TaSP1* but *TaWRKY115^Hap1^* could escape the inhibition by *TaSP1* due to a lack of the 15-bp insertion, therefore, the wheat accessions carrying *TaWRKY115^Hap1^* showed strong cold tolerance. It suggests that *TaWRKY115^Hap1^* as a superior allele could potentially be used for improving cold tolerance in wheat breeding programs.

TFs affect the growth and development of plants not only by activating or suppressing the downstream gene alone but also by interacting with other TFs. For example, OsSHI1 increased the tiller number of rice by negatively regulating the transcriptional activation of IPA1 on *OsTB1* and *OsDEP1* [39]. WRKY-TF interactions have been shown to play an important role in plant response to abiotic stress [40]. In rice, OsWRKY76 interacted with OsbHLH148, activating the expression of *OsDREB1B* to enhance cold tolerance [41]. Therefore, further dissection of the molecular mechanisms of WRKY-protein interactions will provide new insights into better understanding of the complex regulatory network in plants. In recent years, there have been some reports on WRKY-MYB interactions in plants. In grapevine, VvWRKY8 interacted with VvMYB14 to control resveratrol biosynthesis [42]. PtrWRKY19 interacted with PtrMYB074 to form a dimer that is necessary to activate *PtrbHLH186*, thus influencing secondary xylem development in *Populus trichocarpa* [43]. In potato, MYB168 interacted with WRKY20 to regulate lignin monomer synthesis [44]. However, little is known about the regulatory mechanisms of the WRKY-MYB interactions in response to plant abiotic stress. Here, we reported that TaWRKY115 directly interacted with TaMYB4 physically and indirectly influenced *TaCBF12d* expression, which provided new insights into the CBF pathways of wheat in response to low temperature.

C-repeat/DREB binding factor (CBF) has been proven to play an important role in the cold tolerance of plants [33]. *MYB* could regulate the expression of *CBF* genes, resulting in a change in plant cold response [45,46]. Meanwhile, *MYB* was regulated by other genes. In tomato, *HY5* (a bZIP TF) activated the expression of *MYB15* and thereby indirectly affected the expression of CBF genes [47]. In *Arabidopsis*, MPK6 could phosphorylate MYB15 and reduce transcriptional repression of the *CBF3* gene [48]; MYB15 is also ubiquitinated by PUB25 and PUB26 [49]. In rice, *OsMYB30* negatively regulated cold tolerance by suppressing the *BMY* genes via interaction with OsJAZ9 [50]. In this study, we found that TaWRKY115 interacted with TaMYB4, suppressing the expression of *TaMYB4* at both transcriptional and protein levels, thereby alleviating the inhibition of TaMYB4 on *TaCBF12d* expression and finally enhancing wheat cold tolerance. Therefore, we proposed a working model that TaWRKY115-TaMYB4 regulated wheat cold tolerance through involvement in the TaCBF pathway. Notably, in the present study, we only selected *TaCBF12d* as a representative member of the TaCBF family to verify its regulatory relation via the TaWRKY115-TaMYB4 model. However, we also found that the promoter sequences of other six differentially expressed TaCBF members (*TaCBF6a, TaCBF6b, TaCBF6d, TaCBF5d, TaCBF10a* and *TaCBF14d*) contained MYB binding sites and showed similar expression patterns to *TaCBF12d*, possibly suggesting that TaMYB4 simultaneously regulated multiple *TaCBF* genes to affect wheat cold tolerance. In addition, we analyzed the transcriptome profiles for *TaWRKY115* and *TaMYB4* mutants before and after cold treatment via RNA sequencing. GO enrichment analysis showed that DEGs were mainly enriched in lipid transport and metabolism pathways, suggesting that the TaWRKY115-TaMYB4 model may have multiple ways to regulate wheat cold tolerance (Fig. S16). In general, we conceived a *TaSP1-TaWRKY115-TaMYB4-TaCBF* cascade underlying the molecular mechanism of wheat cold tolerance and provided valuable cold tolerance genes for wheat breeding programs.

In this study, we analyzed a geographical distribution of different *TaWRKY115* haplotypes, and found that *TaWRKY115^Hap1^*(cold tolerance haplotype) was distributed across Chinese wheat regions but exhibited a relatively low percentage (<40%) in all wheat test regions of China (Fig. S17A). Further analysis revealed that the percentages of *TaWRKY115^Hap1^* were 12.1% in landrace cultivars, 24.9% in historical cultivars, 24.5% in modern cultivars, 29.1% in introduced cultivars and 32% in synthetic wheat (Fig. S17B). These results suggest that *TaWRKY115^Hap1^* has been artificially domesticated but still shows a big potential application value in wheat breeding due to its relatively low percentage. We also detected a wheat cultivar Jimai816 (JM816) with both favorable haplotypes of *TaWRKY115^Hap1^* and *TaMYB4^Hap3^* (Fig. S17C), and JM816 exhibited strong cold tolerance in the field, thereby providing a valuable germplasm for improving cold tolerance in wheat breeding programs.

## MATERIALS AND METHODS

Plant materials, investigation of CRI in the field, genome-wide association study, RNA-Seq analysis, DAP-seq analysis, EMS mutants, genetic transformation, cold stress treatment and determination of physiological index are described in the Supplementary Data. Detailed procedures of qRT-PCR, subcellular localization in wheat protoplast, Y2-H assay, Pull-down assay, Co-IP assay, LCI assay, ubiquitination assay, Dual-LUC assay, EMSA assay, ChIP-PCR, BSMV-VIGS assay, MST assay and statistical analysis are also provided in the Supplementary Data.

## Supplementary Material

nwag087_Supplemental_File
