# Supplementary material for: TaWRKY115 enhances cold tolerance by weakening the expression of TaMYB4 on CBFs in common wheat
Source: Natl Sci Rev. 2026 Feb 9;13(10):nwag087. doi: 10.1093/nsr/nwag087 (PMC13248867; doi:10.1093/nsr/nwag087)
Supplement: nwag087_Supplemental_File [file nwag087_supplemental_file.docx]

**Materials and Methods**

**Plant materials**

A total of 1430 wheat accessions, including 257 landrace cultivars, 281 historical cultivars, 534 modern cultivars, 334 synthetic wheat and 24 introduced cultivars, were planted at Yuanyang Scientiﬁc Research and Education Center (35.04°N, 113.94°E) of Henan Agricultural University during the 2017-2018 and 2020-2021 cropping seasons [10]. Each accession was planted in two rows. The distance between neighboring plants in each row was 10 cm. Local management was performed for all test accessions. In Yuanyang, the field temperature is around -4ºC ~ -2ºC from the middle of December of 2021 to the middle of February of 2022 (Figure S18).

**Investigation of cold response index in fields**

The cold tolerance of the 233 modern wheat accessions from Yellow and Huai Valley of China was investigated in February and March of each year. The cold response index (CRI) was evaluated based on the criteria of the Wheat Cultivar Approval Committee of the Yellow and Huai valley of China (NY/T2283-2012) by the Ministry of Agriculture and Rural Affairs of China with minor modifications. Generally, the CRI was classified into four levels of 0~3 based on the overall performance of wheat plants (two lines per accession) (Figure S1A), i.e., 0 (cold tolerant), less than 1/5 damaged leaves and no occurrence of dead shoots or dead plants; 1 (moderately tolerant), 1/5 to 1/2 dry leaves and the shoot death rate of <5%; 2 (moderately sensitive), 1/2 to 3/4 dry leaves and shoot death rate of 5%~20%; 3 (sensitive), almost completely dried leaves, the withered leaves accounting for more than 3/4, the visible wrinkled leaf sheaths and the shoot death rate of 20%~60% [9,10].

**Genome-wide association study**

The 233 wheat accessions we scored for CRI were genotyped using the Wheat 660K SNP array as we previously reported [51,52]. Quality preprocessing of the genotyping data was conducted for the SNP call rate (--maf 0.02) and minor allele frequency (MAF) (--geno 0.1) was implemented in PLINK software [53]. The population structure was assessed using STRUCTURE v2.3.4 with unlinked markers (*r*^2^ = 0) [54]. GWAS was performed using the mixed linear model (PCA+K) implemented using the GAPIT packages in *R* software [55]. The *p*-value threshold was set to 1.0e^-3^ for clarifying significant SNPs [54].

**RNA-Seq analysis**

The two-leaf stage seedlings of wheat cultivars YN268 (used for candidate gene screening) were treated at -4ºC for 24 h and 48 h, while two-leaf stage seedlings of Fielder (used for CBF genes analysis), *TaWRKY115-OE* lines and Fielder were planted at normal conditions, *TaWRKY115* mutants (K2716 and K4061) and *TaMYB4* mutant (K4320) were treated at -2ºC for 12 h. Then leaves before and after cold stress were also collected separately to construct RNA pools. All samples were performed with three biological replications. For the *TaWRKY115* and *TaMYB4* EMS mutants, the intersection of differentially expressed genes (DEG) before and after cold treatment were used for GO enrichment synthetically. The cDNA library was constructed and sequenced on a HiSeq 2000 sequencer (Illumina Inc., USA). All of the paired-end reads were mapped to the wheat Chinese Spring genome (IWGSC Ref Seqv1.0) using Bowtie2 v2.2.3 software. Significantly differentially expressed genes were identified with expression level fold change > 2 and FDR < 0.05 [56].

**DAP-seq analysis**

The DAP-seq was conducted to identify genome-wide protein-DNA interactions, which was followed by Bluescape (Beijing, China). The protocol was followed as previously described [57]. Briefly, GST-TaWRKY115 purified protein was combined with the genomic DNA library in *vitro* to further separate out all the DNA that bound to TaWRKY115. Subsequently, the binding sites and possible genes of TaWRKY115 were identified using high-throughput sequencing technology.

**EMS Mutants**

The tetraploid mutants of *TaWRKY115* and *TaMYB4* used in this study were screened from the EMS-mutagenized durum wheat cultivar Kronos library. Two *WRKY115* mutant lines (K2716 and K4061, with a premature stop codon of *WRKY115*) and one *TaMYB4* mutant line (K4320, with a premature stop codon of *MYB4*) were screened to use for functional verification.

The hexaploid mutants of *TaSP1* and *TaMYB4* used in this study were screened from the EMS-mutagenized common wheat cultivar Fielder library. One *TaSP1* mutant line (F7281, with a premature stop codon of *TaSP1*) and one *TaMYB4* mutant line (F9469, with a premature stop codon of *TaMYB4*) were screened to use for functional verification. The hexaploid mutant of *TaCBF12d* used in this study were screened from EMS-mutagenized common wheat cultivar Jimai38 (JM38) library. One *TaCBF12d* mutant line (J5200, with a premature stop codon of *TaCBF12d*) was screened to use for functional verification.

**Genetic transformation**

The coding sequence (CDS) of *TaWRKY115* was amplified from Chinese Spring cDNA and subsequently cloned into the LGY-OE3 vector driven by the Ubi promoter. The LGY-OE3-*TaWRKY115* recombinant plasmid was introduced to the *Agrobacterium tumefaciens* EHA105 strain and was subsequently transferred into immature embryos of wheat cultivar Fielder by Agrobacterium-mediated infection to obtain the *TaWRKY115-OE* plants. Specific primer sets were used to screen putative T_0_ generation of transgenic plants (Table S12). The positive T_0_ plants were self-crossed twice in the greenhouse, and homozygous T_2_ generation lines with high expression were selected for further analysis.

For the construction of gene editing vector, the editing sequences were selected using CRISPR direct (http://crispr.dbcls.jp/). Based on homology search against the genome of Chinese Spring, two sgRNAs (79-98 bp and 199-218 bp in Figure 2C1) were designed in exon 3 of *TaWRKY115*. The target fragment was amplified and inserted into the plGYE-3 vector with the help of the intermediate vector pMETaU6.1. Subsequently, the recombinant plasmid was transferred into Fielder to create the *TaWRKY115*-edited plants. The *TaWRKY115*-edited sites of wheat plants were confirmed through sequencing by Hi-TOM [58]. The *TaWRKY115*-edited plants were self-crossed twice in the greenhouse to obtain T_2_ generation lines.

**Cold stress treatment and determination of physiological index**

The wheat seeds were soaked in 10% H_2_O_2_ for 30 min, and subsequently washed with sterile water. The sterilized seeds were placed in culture dishes with double distilled H_2_O for germination at 25°C. When the coleoptiles of the seeds were 2 cm long, they were transplanted into round plastic pots containing sterilized nutrient soil with a complete nutrient solution. The seedlings were cultured in the greenhouse for about two weeks, and then moved into the illumination incubator for cold stress treatment. According to previous study, hexaploid wheat is generally more tolerant to cold stress than tetraploid wheat [59]. Therefore, when subjected to low-temperature treatment, the temperature for hexaploid wheat was set to -4°C, and the temperature for tetraploid wheat was set to -2°C. The relative electrolyte leakage and relative water content were determined with three replicates for each sample according to our previous method [10]. The proline content was analyzed using the Solarbio Proline Content Assay Kit (BC0290), following the manufacturer's instructions. Chlorophyll fluorescence parameter F_V_/F_M_ ratios (variable fluorescence/maximum fluorescence) as a sensitive indicator was measured using a modulated fluorimeter (PAM-2500, Walz, Germany) according to a previous method [60].

**Quantitative real time -PCR (qRT-PCR)**

The qRT-PCR analysis was performed as described [27] with minor modifications. Total RNA was extracted using TRIZOL reagent (YEASEN, 19201ES60) and purified using RNAclean Kit (TIANGEN, DP412). First-strand cDNA was synthesized using a PrimeScript^TM^ RT Reagent Kit with gDNA Eraser (TaKaRa, RR047A). Relative mRNA levels were detected using a CFX96 Real Time System (Bio-Rad). qRT-PCR was carried out using SYBR Green Master Mix (YEASEN, 11203ES03). The wheat *β*-actin gene (GenBank accession no. AB181991) served as an internal control. The primers used for qRT-PCR are listed in Table S12. Relative expression levels were evaluated according to the relative quantification method ($2^{-\Delta\Delta C_{T}}$) in previous study [61].

**Subcellular localization**

The CDS sequences without stop codons of *TaWRKY115* and *TaMYB4* were cloned into the pJIT163-Ubi-hGFP: GFP expression vector driven by the Ubi promoter using the ClonExpress^®^ II One Step Cloning Kit (Vazyme, C112). The fusion constructs TaWRKY115-GFP and TaMYB4-GFP, and empty vector were introduced into wheat leaf protoplasts via polyethylene glycol-mediated transformation. Then GFP signal was visualized using a Zeiss LSM710 confocal laser microscope after a 12 h incubation in the dark.

**Yeast two-hybrid (Y2-H) assay**

The CDS of *TaWRKY115* was cloned into the pGBKT7 vector as the bait construct. The interacting proteins were screened from a cDNA library of Jiyanmai 7 containing multiple tissues according to the manufacturer's protocol (Clontech, PT3024-1). The full-length CDS and various truncated versions of *TaMYB4* were amplified and subcloned into the pGADT7 vector to create the prey vector. Then the bait and prey vectors were co-transformed into yeast strain AH109 by the polyethylene glycol-mediated method, according to the Matchmaker^®^ Gold Y2H System user manual. After transfection, the transfected cells were cultured on SD/-Leu/-Trp media to screen the positive transformants, then the positive colonies were further verified on SD/-Leu/-Trp/-His+AbA+X-α-Gal and SD/-Leu/-Trp/-His/-Ade+AbA+X-α-Gal selective medium to check the interaction.

**Pull-down assay**

The plasmids of GST-TaWRKY115, His-TaMYB4 and GST were transformed into *Escherichia coli* BL21 (DE3), respectively. Recombinant protein was expressed after induction 0.2 mM isopropyl *β*-D-1-thiogalactopyranoside (IPTG), and was purified using the GST-labeled protein purification kit (Beyotime, P2262) and His kit (Beyotime, P2226) according to the operation manual of companies. Purified His-TaMYB4 protein was incubated with purified GST-TaWRKY115 and GST at room temperature for 30 min, respectively. Next, 50 µL of GST-Trap agarose was introduced into the reaction system, and then was incubated at 4 °C for 6 h [10]. After that, the beads were collected and washed with Tris-buffered saline solution. Subsequently, the washed beads were boiled with loading buffer and then analyzed by Western blot using anti-GST antibody (PTM Biolabs, PTM 5046) and anti-His antibody (Abmart, M30111). His-TaMYB4+GST was used as the negative control.

**Co-IP assay**

The plasmids of 0.2 mg 35S:TaMYB4-FLAG with 0.2 mg 35S:TaWRKY115-GFP or 0.2 mg empty 35S vector were co-transformed into 2 mL of protoplasts derived from the wheat seedlings and were incubated at 25°C for 12 h. After collecting the cells by centrifugation at 4°C and 100 g for 2 min, we feezed them in liquid nitrogen and then resuspended and lysed with 1 mL of lysis Buffer (20 mM Tris-HCl pH8.0, 2 mM DTT, 1% Triton X-100, 100 μM PMSF, 250 mM sucrose), and the supernatant was used as the Input. Next, 10 µL of GFP-Trap magnetic beads were mixed with the supernatant by vertical rotation at 4°C for 2 h. After washing with PBS, the GFP-TaWRKY115 or GFP protein bound to the GFP-Trap magnetic beads was eluted by heating at 95°C for 10 min, and the protein separated from the magnetic beads was used for IP. After SDS-PAGE separation of the Input and IP samples, anti-GFP (Abcam, ab13970) and anti-Flag antibodies (Abmart, M2008L) were used for immunoblot analysis.

**Firefly luciferase complementation imaging (LCI) assay**

The CDS sequences of *TaWRKY115* and *TaMYB4* were inserted into pCAMBIA1300-cLUC and pCAMBIA1300-nLUC, respectively, to generate the fusion constructs cLUC-TaWRKY115 and nLUC-TaMYB4. Then the constructs were separately transformed into *Agrobacterium* strain GV3101. Different vector combinations were co-infiltrated into 4-week-old *N. benthamiana* leaves using a needleless syringe. Empty vectors expressing cLUC or nLUC were co-transformed as the negative controls. After 2 days of incubation in darkness, the injected leaves were harvested and incubated with 1mM luciferin (Promega, E1605), and the luminescence images were captured using a CCD imaging system (Berthold, LB985).

**Ubiquitination assay**

For the ubiquitination assay, total proteins were extracted from Fielder and TaWRKY115-OE plants using lysis buffer (20 mM Tris-HCl, 2 mM DTT, 1% Triton X-100, 100 μM PMSF, 250 mM sucrose; pH8.0) containing a protease inhibitor cocktail. Next, they were incubated with purified TaMYB4-His protein at 25°C for 4 h. The ubiquitination of TaMYB4 was detected via Western blot with anti-Ub (Abcam, United Kingdom, Cat. No. Ab134953, 1:3000). Anti-actin antibody (Abmart, M2009L) and anti-His antibody (Abmart, M30111) were used as controls.

**Dual-Luciferase reporter assay**

The promoter region of *TaCBF12d* (373 bp) containing the MYB binding site, the promoter sequence of *TaMYB4* (2067 bp) and the promoter region of *TaWRKY115* (245 bp) containing TaSP1 binding site were respectively inserted into pGreen0800-LUC as a reporter. The CDS sequences of *TaMYB4*, *TaWRKY115* and *TaSP1* were fused with CaMV35 promoter, respectively, to produce effectors. The constructed effectors and reporter plasmids were co-transferred into leaves of 4-week-old tobacco plants (*N. benthamiana*). After inoculation, plants were immediately covered with lids and incubated in the dark at 25°C for 8 h, and then placed in a controlled growth chamber at 25°C with a photoperiod of 16 h light and 8 h dark. Total protein was extracted from the infected area after culturing for 3 days. For cold treatment, after culturing for 24 h at 25°C, the tobacco was transferred to a 4°C incubator for further cultivation. The fluorescent values of LUC and REN were detected using a Dual-Luciferase Reporter Assay System (Promega, E1910) based on the manufacturer’s manual by GloMax^®^ 20/20 Luminometer (Promega, USA). The value of LUC was normalized to that of REN. At least six transient assay measurements were included for each pair.

For transient expression assays of *TaWRKY115* promoter activity, the promoter fragments with or without 15-bp were constructed into pGREENII 0800-LUC vector, respectively. The determination method was the same as above.

**Transient expression assays**

To examine whether TaWRKY115 could affect the expression of TaMYB4, the 35S:TaMYB4-GFP-FLAG and 35S:TaWRKY115-GFP or 35S:GFP were injected into four-week-old tobacco leaves. To investigate the pathway of TaWRKY115 degraded TaMYB4, after 24 h inoculation, 50 µM MG132 and 10 mM MgCl₂ were injected. For cold treatment, after culturing for 24 h at 25°C, the tobacco was transferred to a 4°C incubator for further cultivation. After 48 h, the tobacco leaves were collected and stored in liquid nitrogen. Total proteins were extracted in lysis buffer (20 mM Tris-HCl pH8.0, 2 mM DTT, 1% Triton X-100, 800 μM PMSF, 250 mM sucrose) containing a protease inhibitor cocktail. Proteins were detected by immunoblot using anti-FLAG (Abmart, M2008L). Moreover, the protoplast of Fielder and *TaWRKY115*-overexpressed wheat leaves were extracted and 163hGFP:TaMYB4 was transferred instantly. The protein was extracted and analyzed using an anti-GFP antibody (Abcam, ab13970). An anti-actin antibody (Abmart, M2009L) was used as a control.

**Electrophoretic mobility shift assay (EMSA)**

The 46-bp DNA fragments from the *TaWRKY115^Hap1^* (without GC-box), *TaWRKY115^Hap2^* (with the GC-box GGGCGG), *TaMYB4* (W-box GGATCC regulated by *TaWRKY115*), *TaCBF12d* (MYB-binding motif CAACAG regulated by *TaMYB4*) labelled with biotin were synthesized to use as the probes, respectively. Then EMSA was performed using a LightShift Chemiluminescent EMSA Kit (Thermo Scientific, 20148) according to the manufacturer's instruction. The biotin-labelled probe was incubated in a reaction mixture (1×binding buffer, 2.5% glycerol, 50 mM KCl, 5 mM MgCl_2_, and 10 mM EDTA) with TaSP1, TaWRKY115 and TaMYB4 protein at room temperature for 20 min, and free bound DNA fragments were separated by acrylamide gel electrophoresis. To test the binding specificity, the cold probe (unlabeled probe), mutated probe (mutated and labeled probe, with the GC-box, MYB-binding site or W-box sequences changed to AAAAAA), and mutated cold probe (unlabeled and mutated probe) were used as controls.

**ChIP-PCR assay**

ChIP assays were performed according to [62] with some minor modifications. The 35S empty vector and *35Spro:TaMYB4-GFP* recombinant vector were transferred into wheat protoplasts. After incubation in the dark for 18 h, protoplasts were collected and stained in a 1% formaldehyde solution immediately. The target protein was cross-linked with DNA. The chromatin DNA was purified by isolating the nuclei and then sheared into fragments using sonication. The protein-DNA complex was incubated with an anti-GFP antibody (Abcam, ab13970). After extensive washing and de-crosslinking, the precipitated and input DNA samples were analyzed by PCR amplification and gel electrophoresis using specific primers (Table S12).

**Virus-induced gene silencing (VIGS) assay**

The VIGS assay was conducted according to previous report [63]. Wheat cultivar YN268 was selected for the VIGS experiment. The 167 and 180 bp fragments for *TaMYB4* and *TaCBF12d* were cloned and then inserted into the γ vector, respectively. The constructs were linearized and used to synthesize α, β, γ RNAs of the BSMV genome using RiboMAX^TM^ Large Scale RNA Production System-T7 (Promega, P1300). The in vitro transcripts of each RNA segment were mixed in equal amounts and diluted with an equal volume of RNAase-free water, and added to FES buffer. The BSMV_0_ that constructed by α, β and γ RNA derived from the original empty vector pSL038-1 was used as the virus negative control, while BSMV: PDS was used as the positive control. The BSMV RNAs were synthesized in vitro and rub-inoculated onto the 2nd fully expanded leaf of wheat seedings at 3-leaf stage. All the seedlings were cultivated in an artificial climate chamber at 23°C, with darkness for 24 h, followed by a 16h/8h light/dark photoperiod. The silencing efficiency was detected by qRT-PCR from the 3rd and 4th leaves (about 0.2~0.3 g) of each treatment group at 14 days post-inoculation.

**Micro-scale thermophoresis (MST) assay**

The interaction between TaWRKY115 and TaMYB4 was using the Monolith NT.115 instrument (Nano Temper Technologies, Munich, Germany). Briefly, the purified TaWRKY115 was labelled using the Monolith NT protein labeling kit according to the manufacturer's instructions, and then mixed with purified TaMYB4 or OsWRKY76. After incubation, the mixed protein samples were loaded into the capillaries and analyzed using the Nano Temper analytical software to determine the equilibrium dissociation constant (KD) according to previous study [64].

MST assay was also used to analyze the interaction between TaMYB4 and *TaCBF12d* promoter, and the process is similar to the above.

**References**

1. Yang X, Zhang L, Wei J, et al. (2025) A TaSnRK1α-TaCAT2 model mediates resistance to Fusarium crown rot by scavenging ROS in common wheat. *Nat Commun*. **16,** 2549.
2. Niaz M, Zhang L, Lv G, et al. (2023) Identification of *TaGL1-B1* gene controlling grain length through regulation of jasmonic acid in common wheat. *Plant Biotechnol J*. **21**, 979-989.
3. Purcell S, Neale B, Todd–Brown K, et al. (2007) PLINK: a tool set for whole–genome association and population–based linkage analyses. *Am J Hum Genet*. **81**, 559-575.
4. Sun C, Zhang F, Yan X, et al. (2017) Genome-wide association study for 13 agronomic traits reveals distribution of superior alleles in bread wheat from the Yellow and Huai Valley of China. *Plant Biotechnol J*.**15,** 953-969.
5. Barrett J, Fry B, Maller J, et al. (2005) Haploview: analysis and visualization of LD and haplotype maps. *Bioinformatics*. **21**, 263-265.
6. Zhao L, Yang Y, Hu P, et al. (2023) Genetic mapping and analysis of candidate leaf color genes in common winter wheat (*Triticum aestivum* L.). *Mol Breed*. **43,** 48.
7. Su H, Cao L, Ren Z, et al. (2024) ZmELF6-ZmPRR37 module regulates maize flowering and salt response. *Plant Biotechnol J*. **22,** 929-945.
8. Liu Q, Wang C, Jiao X, et al. (2019) Hi-TOM: a platform for high-throughput tracking of mutations induced by CRISPR/Cas systems. *Sci China Life Sci*. **62**, 1-7.
9. Nejadsadeghi L, Maali-Amiri R, Zeinali H, et al. (2014) Comparative analysis of physio-biochemical responses to cold stress in tetraploid and hexaploid wheat. *Cell Biochem Biophys*. **70**, 399-408.
10. Feng W, Qi S, Heng Y, et al. (2017) Canopy Vegetation Indices from *In situ* Hyperspectral Data to Assess Plant Water Status of Winter Wheat under Powdery Mildew Stress. *Front Plant Sci*. **8,** 1219.
11. Hou W, Lu Q, Ma L, et al. (2023) Mapping of quantitative trait loci for leaf rust resistance in the wheat population 'Xinmai 26/Zhoumai 22'. *J Exp Bot*. **74,** 3019-3032.
12. Ifnan M, Zhang Y, Liu Z, et al. (2018) *CaWRKY40b* in Pepper Acts as a Negative Regulator in Response to Ralstonia solanacearum by Directly Modulating Defense Genes Including *CaWRKY40*. *Int J Mol Sci*. **19**, 1403.
13. Lv G, Zhang Y, Ma L, et al. (2023) A cell wall invertase modulates resistance to fusarium crown rot and sharp eyespot in common wheat. *J Integr Plant Biol*. **65,** 1814-1825.
14. Liu P, Shi C, Liu S, et al. (2023) A papain-like cysteine protease-released small signal peptide confers wheat resistance to wheat yellow mosaic virus. *Nat Commun*. **14,** 7773.
